# Shedding Light on Pupillometry Shadows: Understanding Opioid Effects on Pupillary Metrics in ICU Monitoring

**DOI:** 10.1111/aas.70085

**Published:** 2025-06-25

**Authors:** Luis Schulz, Anders Aneman

**Affiliations:** ^1^ Intensive Care Unit Liverpool Hospital, South Western Sydney Local Health District Sydney New South Wales Australia; ^2^ Southwestern Clinical School University of New South Wales Sydney New South Wales Australia

In recent years, automated pupillometry has become an important addition to the clinical toolbox of the intensivist. Traditionally based on subjective clinical observation, pupillary assessment has long been impaired by inter‐observer variability [1], inconsistent stimulus delivery, and the inherent limitations of the human eye in measuring minute changes in pupil responses. The introduction of computer‐assisted pupillometry—bringing quantifiable, objective, and reproducible measurements—undoubtedly represents a welcome advance in neuromonitoring.

This technology has received particular attention in the setting of neuroprognostication following Traumatic Brain Injury [2] and cardiac arrest [3, 4, 5], being increasingly suggested in guidelines for its potential to provide standardized assessments of brainstem function [6, 7]. Pupillary size and reactivity—previously assessed with nothing more than a penlight and clinical intuition (with age bringing valuable experience, but often at the expense of visual acuity)—were to be described in vague terms like “sluggish.” Today, these can be quantified with high precision using infrared sensors and computer vision algorithms, offering a quantitative, extended assessment, including new metrics with the analysis of velocity. In particular, the speed of contraction and, importantly, dilation—reflective of sympathetic and parasympathetic balance—can thus be monitored in real‐time, offering a more granular understanding of neurological status.

However, the enthusiasm for automated pupillometry must be tempered by a careful examination of its limitations and the transparency of its metrics. Pupillometry, specifically using the Neurologic Pupil Index (NPi) has been described as being less susceptible to the confounding effects of sedation and analgesia [8, 9]. This claim, while appealing, is derived from relatively limited data and warrants closer scrutiny. The NPi is a proprietary, dimensionless score derived from comparison to a normative sample of undisclosed characteristics, with its underlying components, computational weights, and exact derivation remaining opaque—raising ethical and scientific concerns. When clinicians are asked to make decisions based on opaque algorithms, the lack of interpretability can undermine both the clinical and moral integrity of care. No medical tool should operate as a black box, particularly when used in critical decisions such as prognostication. Alternatively, another commercially available device offers a score derived from a quantitative measurement of the Photomotor Reflex, the Quantitative Pupil Index (QPi). Similar to NPi, QPi aims to simplify clinical usability by condensing complex physiological signals into a single summary value. However, this approach of abstracting the underlying data risks detaching clinicians' reasoning from key pupillary features. In the complex, heterogeneous ICU population, assuming scores or any pupillometric measurement are insulated from pharmacologic or pathophysiological influences is not only simplistic—it may be misleading.

The current study by Sorensen et al. makes an important contribution by specifically examining the impact of opioids on pupillary dilation velocity in ICU patients, using ultra‐high‐performance liquid chromatography coupled with tandem mass spectrometry (UHPLC–MS/MS) to accurately quantify opioid levels. By identifying a dose‐dependent relationship and potentially differential agent‐specific effects, the study offers critical insights into how analgesia can influence a key pupillometric parameter. This is an important step forward in contextualizing pupillometry data and recognizing the pharmacodynamic terrain within which these measurements are obtained.

As the field of critical care continues to embrace technologies that promise objectivity and precision, it becomes even more important to ensure that these tools are transparent, interpretable, and empirically grounded. Automated pupillometry is indeed a promising modality, but its metrics must be demystified, and its vulnerabilities clearly understood. Future research should move beyond reliance on composite indices alone and focus on systematically analyzing the individual physiological parameters that underlie pupillary responses. Meaningful advancement in critical care monitoring depends not on how easily we can use a tool, but on how deeply we understand what it measures.

## Author Contributions

L.S. was involved in writing, review and editing. A.A. was involved in writing, review and editing.

## Conflicts of Interest

The authors declare no conflicts of interest.

## Data Availability

Data sharing not applicable to this article as no datasets were generated or analyzed during the current study.
